# Guiding flow of light with supersymmetry

**DOI:** 10.1038/s41377-022-00988-1

**Published:** 2022-10-06

**Authors:** Can Huang, Qinghai Song

**Affiliations:** 1 Ministry of Industry and Information Technology Key Lab of Micro-Nano Optoelectronic Information System, Harbin Institute of Technology, Shenzhen, 518055 China; 2 Pengcheng Laboratory, Shenzhen, 518055 China; 3 Collaborative Innovation Center of Extreme Optics, Shanxi University, Taiyuan, Shanxi 030006 China

**Keywords:** Silicon photonics, Nanophotonics and plasmonics

## Abstract

The continuous supersymmetry transformation is applied to the silicon waveguides, and the guidance and conversion of any mode in a wide spectral range are successfully realized in experiments. This proves its great potential in optical spatial mode modulation and space division multiplexing in optical communication.

For thousands of years, the study of light propagation has profoundly affected the progress of human civilization. Early in ancient Greece, people found when light beam incident from air into water, its propagation direction changed. Later, people discovered the law of refraction and designed practical tools such as lenses and telescopes. In order to control the behavior of light propagation more accurately, human have always been trying to change the rules of light propagation. In recent years, people have proposed utilizing transformation optics and metamaterials to realize some fancy functions such as invisibility cloak^1,2^. However, the transformation optics based on coordinate transformation often produces extreme material parameters, and the devices can only work in a limited band. Therefore, it is necessary to demonstrate a new paradigm to break through the limitations of traditional transformation optics. In a recent article published by eLight^3^, Yim et al. proved in experiment that controlling and switching multiple optical beams in broadband scope can be achieved at the same time using the principle of supersymmetry (SUSY).

The basic idea of supersymmetric transformation optics originally comes from quantum field theory, in which people can treat bosons and fermions equally by introducing supersymmetric transformation^4,5^. Due to the similarity between the Schrodinger equation and the optical paraxial wave equation, people also try to use the mathematical principle of SUSY in optical design^6,7^. The main idea of SUSY transform optics is to construct a super-partner system of a known optical system. The SUSY is regarded as “not broken” when the super-partner system lacks the eigenvalues of a state, but possesses the eigenvalues of the other states in the original system. On the contrary, the SUSY is “broken” if the super-partner system and the original optical system possess exactly the same eigenvalues. In SUSY optical system, the original optical structure and the super-partner structure can generally achieve global phase coupling, then many novel physical phenomena can be realized through strategic design. For a known discrete optical system without SUSY breaking, the super-partner structure can be constructed by the Cholesky method or QR decomposition. Using the principle, people can design desirable optical structures, such as photonic lattices with the same scattering characteristics^8^, on chip mode converter^9^, and the high-power single-mode laser array^10–12^ etc.

However, for some more complex application scenarios, SUSY transformation requires the optical potential distribution to be continuous, and its mathematical framework is proved to be a continuous transformation. Although the realizability of this principle has been introduced in the previous theoretical work^13^, the experimental proof has not been reported. In the paper reported by Yim et al.^3^, they proposed and designed gradient-index(GRIN) optical structures on the silicon-based platform to realize continuous SUSY transformation (Fig. 1). In the experiment, the gradient-index material can be realized by adjusting the filling ratio of Si (i.e., the size of the air gap). They also showed how to use the designed optical structure to realize the routing, switching and spatial mode shaping in a broadband scope (1460–1570 nm). The transmission measurement also show that the system can ensure the high transmittance of the beams with negligible crosstalk between neighboring channels while realizing these functions, thus showing the advantages of SUSY optical system over traditional metamaterial optical system.

From a fundamental point of view, this work experimentally shows the interplay of supersymmetry and a metamaterial can contribute to the increase of the spatial degree of freedom in integrated photonics design. This breakthrough discovery may affect a wide range of scientific fields, including optics, integrated photonics, optical communications, and quantum physics, etc. From a practical point of view, future researches need to consider how to quickly realize dynamic switching to realize arbitrary steering and routing of the flow of light with SUSY transformation . Moreover, whether this platform can realize the functionalities demonstrated here in single photon level or other quantum information processing needs further research.

**Fig. 1:**
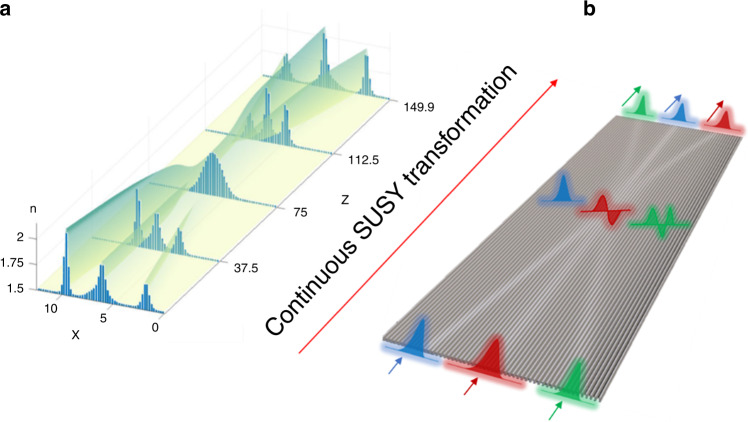
Continuous SUSY transformation optics and its demonstration on a Si chip. **a** Two dimensional refractive index distribution n(x, z) designed by SUSY transformation (unit: um); **b** GRIN metamaterial designed to substantiate n(x, z), where different optical states (blue, red, green) with different propagation constants can propagate with spatial characteristics and directions dictated by the SUSY transformation
